# Cholera in Nashville—Relation to Limestone

**Published:** 1849-03

**Authors:** 


### CHOLERA IN NASHVILLE—RELATION TO LIMESTONE.

We have just received a letter from Dr. H. B. Walton, of Nashville, giving some interesting particulars in relation to the appearance of cholera in that city. He states that the pestilence first appeared there about two months since, and that the average mortality from it had been about two a day. "The weather for the greater part of the time has been warm and wet. But," he adds, "the point to which I wish particularly to call your attention is the predilection of the disease for a certain quarter of the city. It has prevailed almost exclusively about a particular locality. At first, this appeared inexplicable; but since reading the remarks of Dr. Jackson on the connection between cholera and limestone regions, I have supposed that the cause was revealed. A large portion of the city of Nashville is supplied with water from the Cumberland; citizens in other parts use water from springs and wells which, of course, is largely impregnated with carbonate of lime. It is to the latter, with scarcely an exception, that cholera has been confined. Two cases presented themselves which, at first, I supposed, were exceptions to the rule. One was a lady, who resided in that portion of the city where hydrant water is used; the other was a negro man living in the same quarter; but, on inquiry, I learned that the former used water from a spring in the cellar of her dwelling, and that the latter had been laboring in the vicinity of a spring from which he obtained all the water that he drank.

"A large majority of the more aggravated cases have occurred in a small neighborhood, in the vicinity of a spring more highly charged with lime than any other in the city. Whether it is to the use of this water, or to some other cause, that the disease has prevailed in this locality while the city has been exempt from it, is a question not to be determined without farther observation. But the fact is instructive, and, as bearing upon the local origin of cholera, I have deemed it worthy of being recorded."

It may be interesting to remark, in reference to the suggestion of Dr. Walton, that *cholera infantum* has prevailed with much less severity in Nashville since the introduction of hydrant water, as we were assured by physicians there some years ago. Nevertheless, we are not prepared to give our assent to the doctrine that cholera is produced by drinking limestone water. That it manifested a preference for limestone districts, in its former visit to our country, we had occasion to remark two years since, when speaking of the bearing of geology upon disease; but it does not follow that the water had any connection with it. We attempted to show that it was particularly in regions where the older, or blue limestone is the surface rock, that the pestilence was most fatal. But this is not more soluble than the other limestones. The water at Louisville is as highly charged with the grey limestone, as the water at Lexington is with the blue. In Louisville, the epidemic at its height carried off seven a day, in a population of 20,000; in Lexington, in a population of 5,000, sixty persons died in one day. At New Orleans, where there is no limestone, the disease was excessively destructive. At Cincinnati, Maysville, Lexington, and Versailles, all on the blue limestone, its mortality was great. At Nashville, again, at Murfreesborough, Shelbyville, and Pulaski, it appeared in a malignant form, and they are all upon this rock. A few miles south of the latter places, the formation changes, and the epidemic was heard of no where beyond them. These facts are curious. They seem to show a relation between cholera and our geological formations; but they do not reveal the cause of the pestilence.

